# Resting-state functional connectivity alterations in periventricular nodular heterotopia related epilepsy

**DOI:** 10.1038/s41598-019-55002-3

**Published:** 2019-12-05

**Authors:** Wenyu Liu, Xinyu Hu, Dongmei An, Dong Zhou, Qiyong Gong

**Affiliations:** 1 Departments of Neurology, West China Hospital, Sichuan University, No. 37 GuoXue Alley, Chengdu, 610041 China; 2 Departments of Radiology, Huaxi MR Research Center (HMRRC), West China Hospital, Sichuan University, No. 37 GuoXue Alley, Chengdu, 610041 China

**Keywords:** Brain imaging, Epilepsy

## Abstract

Periventricular nodular heterotopia (PNH) is a neural migration disorder which often presents clinically with seizures. However, the underlying functional neural basis of PNH is still unclear. We aimed to explore the underlying pathological mechanism of PNH by combining both whole brain functional connectivity (FC) and seed-based FC analyses. We utilized resting-state fMRI to measure functional connectivity strength (FCS) in 38 patients with PNH-related epilepsy and 38 control subjects. The regions with FCS alterations were selected as seeds in the following FC analyses. Pearson correlation analyses were performed to explore associations between these functional neural correlates and clinical features. In comparison with controls, PNH patients showed lower FCS in bilateral insula (P < 0.05, family wise error (FWE) correction), higher FC in the default mode network and lower FC in the fronto-limbic-cerebellar circuits (P < 0.05, FWE correction). Pearson correlation analyses revealed that FCS in bilateral insula was negatively correlated with the epilepsy duration (P < 0.05); medial prefronto-insular connectivity was negatively correlated with Hamilton Anxiety Scale (P < 0.05) and cerebellar-insular connectivity was also negatively correlated with Hamilton Depression Scale (P < 0.05). Using the resting-state FCS analytical approach, we identified significant insular hypoactivation in PNH patients, which suggests that the insula might represent the cortical hub of the whole-brain networks in this condition. Additionally, disruption of resting state FC in large-scale neural networks pointed to a connectivity-based neuropathological process in PNH.

## Introduction

Periventricular nodular heterotopia (PNH) is a common structural malformation of cortical development in which nodules of neurons are ectopically retained along the lateral ventricles^1^. The clinical manifestations of PNH is characterized by seizure disorder ranging from mild to intractable, mental retardation, hypotonia and reading dysfluency^2^. Multiple lines of evidence demonstrated that the mutations in Filamin A (FLNA) gene is relevant to the genetic basis of this disorder^3^. Although the most common genetic cause of PNH has been identified, the neural correlates of PNH remain elusive.

Advances in modern MRI techniques have dramatically increased our understanding regarding the neural substrates of cortical developmental malformations including PNH^4^. For example, previous whole-brain diffusion MRI tractography revealed anomalous fiber projections in nodular tissue suggestive of abnormal organization of white matter in patients with PNH^5^. One recent diffusion tensor imaging (DTI) study showed significant fractional anisotropy (FA) reductions in the genu and splenium of the corpus callosum in PNH patients compared with normal controls^6^. Christodoulou *et al*. demonstrated that most heterotopic nodules in PNH are structurally connected to overlying cortex, and the strength of abnormal connectivity is higher among patients with the longest duration of epilepsy^7^. Although researches regarding abnormalities of structural anatomy in PNH have achieved remarkable progress, the involvement of functional neural basis in the pathogenesis of PNH received considerably less attention.

During the last few decades, resting-state fMRI has become a promising imaging approach which could be applied to measure abnormalities in spontaneous activity of the brain without performing tasks^8,9^. Functional connectivity (FC), the intrinsic slow (less than 1 Hz) fluctuations in hemodynamics that can be measured in the resting-state fMRI, emerged as a helpful method for reflecting the level of integration of localized activition across different brain areas^10^. Previous rs-fMRI studies have demonstrated that, compared with normal controls, patients with PNH showed higher FC in the prefrontal cortices, supramarginal gyrus, dorsal cingulate gyrus, and lower FC in the parahippocampal gyrus and inferior temporal gyrus^7,11^. However, all of these resting-state fMRI literature adopted seed-based FC approach, which is the most commonly used method to identify the resting state networks. Briefly, seed-based analysis is a model-based method in which we can choose a seed or region of interest (ROI) and find the linear correlation of this seed region with all the other voxels in the entire brain^12^. This approach is useful to discover regionally specific hypotheses of cerebral function but has limited ability to detect connectivity patterns not predicted a priori^13^.

Recently, whole-brain functional connectivity known as functional connectivity strength (FCS) has been recognized as a powerful analytical index of resting-state fMRI to identify the brain areas with high-degree centrality in whole-brain networks^14^. Unlike the traditional seed-based FC approach, there is no need to select a priori seed as ROI in the FCS calculation, which enables the evaluation of aberrant connectivity in each brain area at the whole brain level^15^. Previous evidence has suggested that the FCS metric is related to physiological measures including regional glucose metabolism and cerebral blood flow^16^. Notably, the FCS method has already been applied to investigate the neuropathological mechanisms of several neuropsychiatric disorders^13,17^.

Thus, the aim of the current study was to combine both whole-brain FC and seed-based FC analyses to explore the network-level neural function alterations in patients with PNH using resting-state fMRI. Specifically, we firstly evaluated abnormalities in whole-brain FC, as measured by FCS, in PNH patients compared with controls. Additionally, regions with significant FCS alterations were selected as ROIs in subsequent seed-based FC analyses. Finally, we investigated the correlations between these functional neural substrates and clinical features in PNH patients.

## Results

### Demographic characteristics

The sociodemographic characteristics of all subjects enrolled in this study are displayed in Table 1. Thirty-eight right-handed patients, including 15 males and 23 females, previously diagnosed with PNH were included in the current investigation and were actively contacted for recruitment. In the PNH group the mean age was 27.6 years (range: 9–56 years, standard deviation (SD): 10.7 years). The mean onset age of first seizure was 19.9 years (SD: 8.4 years). Based on the MMSE scores, no patients exhibited cognitive impairment. All patients had also been diagnosed with epilepsy with one patient naïve to any epileptic drugs before involved in this research. Of the remaining patients that had previously used treatment; all were diagnosed with epilepsy, 23 were treated on monotherapy, 14 on polytherapy, and all had a history of focal seizures. 18 of the patients reported having a history of only focal seizures. In response to medical treatments (patient’s response to epilepsy drugs), 15 patients reported being seizure free, and the rest reported having occasional seizures. None of them had any cognitive impairment. They were all diagnosed with PNH after clinically presenting with seizures.

**Table 1:** Demographic information of patients with PNH and HCs.

Characteristics	PNH (n = 38)	HCs (n = 38)	P Value
Age (years)	27.61 ± 10.1	27.50 ± 9.76	0.963^a^
Sex (male/female)	15/23	15/23	1.000^b^
MMSE	27.94 ± 0.89	28.19 ± 1.06	0.284^b^
Age at onset (years)	19.9 ± 8.5	—	—
Duration (years)	7.6 ± 7.5	—	—
Last seizure (months)	10.1 ± 4.3	—	—
Family history ( + /−)	0/38	—	—
Aura ( + /−)	6/32	—	—
Seizure type (focal-only/SGTCS)	18/20	—	—
Number of AEDs (median (range)	1(1–2)	—	—
Treatment timed (years)	6.3 ± 4.8	—	—

Values are mean ± SD.

^a^Chi-square test.

^b^Two-tailed two-sample *t* test.

Thirty-eight age and sex-matched HCs with negative MRI examinations were included for comparison. The mean education years in control group was 11.2 years (SD: 3.5 years). No significant differences in the age, sex, handedness, years of education, and MMSE scores were identified between the PNH patients group and control group (*P* > 0.05) (Table 1).

The mean scores of HAMA and HAMD in patients with PNH were 11.58 ± 6.23 and 13.05 ± 7.16, respectively. In detail, 20 of the patients reported anxiety symptoms (52.63%), of which 13 (65%) experienced probable anxiety, 3 (15%) experienced definite anxiety, and 1 (5%) experienced severe anxiety. 16 (42%) patients reported possible depression symptoms, of which 5 (31%) experienced mild or moderate depression, while none of the patients experienced severe depression symptoms.

### Functional connectivity strength

**Figure 1:**
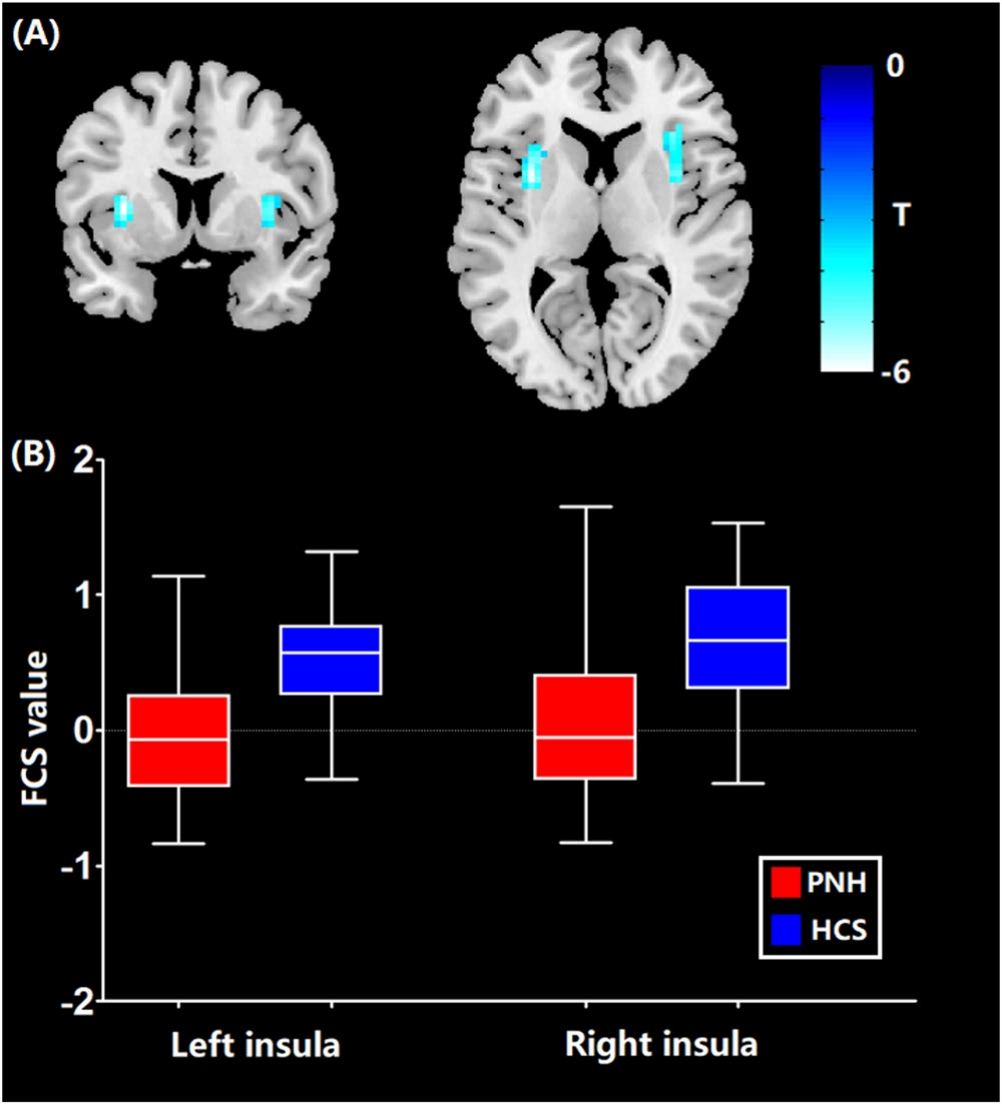
Brain areas with increased FCS in patients with PNH relative to healthy controls. *Abbreviations:* FCS, functional connectivity strength; PNH, periventricular nodular heterotopia.

The differences of functional connectivity strength between PNH patients and controls are shown in Table 2. None of the brain regions examined displayed higher FCS values in the PNH group compared with the controls, while lower FCS in the bilateral insula of PNH patients was observed compared to controls (FWE corrected p < 0.05) (Fig. 1).

**Table 2:** Significant differences of functional connectivity strength between patients with PNH and HCs.

Brain region	Voxels	Peak MNI coordinates			T	P value
		X	Y	Z		
L insula	82	−33	3	9	5.97	0.02
R insula	71	33	24	6	4.76	0.034

### Seed-based FC

**Figure 2:**
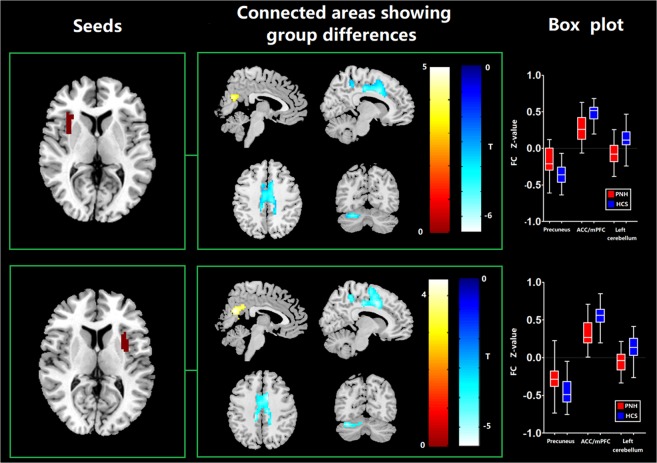
Anatomical replicas of altered FC between PNH patients and healthy controls. *Abbreviations:* FC, functional connectivity; PNH, periventricular nodular heterotopia.

The differences in seed-based FC between PNH patients and controls are shown in Table 3. Compared to controls, the PNH patients showed higher FC in the precuneus and lower FC in the left cerebellum and anterior cingulate cortex (ACC) extending to medial PFC with seed placed in the left insula where increased FCS was detected (p < 0.05, FWE corrected). These findings of FC alterations remained reproducible when the seed was placed in the right insula (p < 0.05, FWE corrected) (Fig. 2). Meanwhile, we found a significant hyper-influence of the left insula over the precuneus of subjects with PNH (210 voxels, T = 4.43, P < 0.001, FWE correction at the cluster level, Fig. S1).

**Table 3:** Significant differences of seed-based functional connectivity between patients with PNH and HCs.

Seed	Connected area	Peak MNI coordinates			Voxel size	T	P value
		X	Y	Z			
**Regions with increased FC in PNH patients relative to HCS**							
L insula	Precuneus	−3	−66	21	194	4.58	0.002
R insula	Precuneus	3	−63	21	223	4.41	0.001
**Regions with decreased FC in PNH patients relative to HCS**							
L insula	ACC/mPFC	9	9	36	467	5.69	<0.001
L insula	L cerebellum	−27	−66	−24	105	5.36	0.028
R insula	ACC/mPFC	15	0	66	498	5.61	<0.001
R insula	L cerebellum	−36	−72	−24	88	5.17	0.049

### Associations between clinical features and altered functional neural correlates of PNH patients

**Figure 3:**
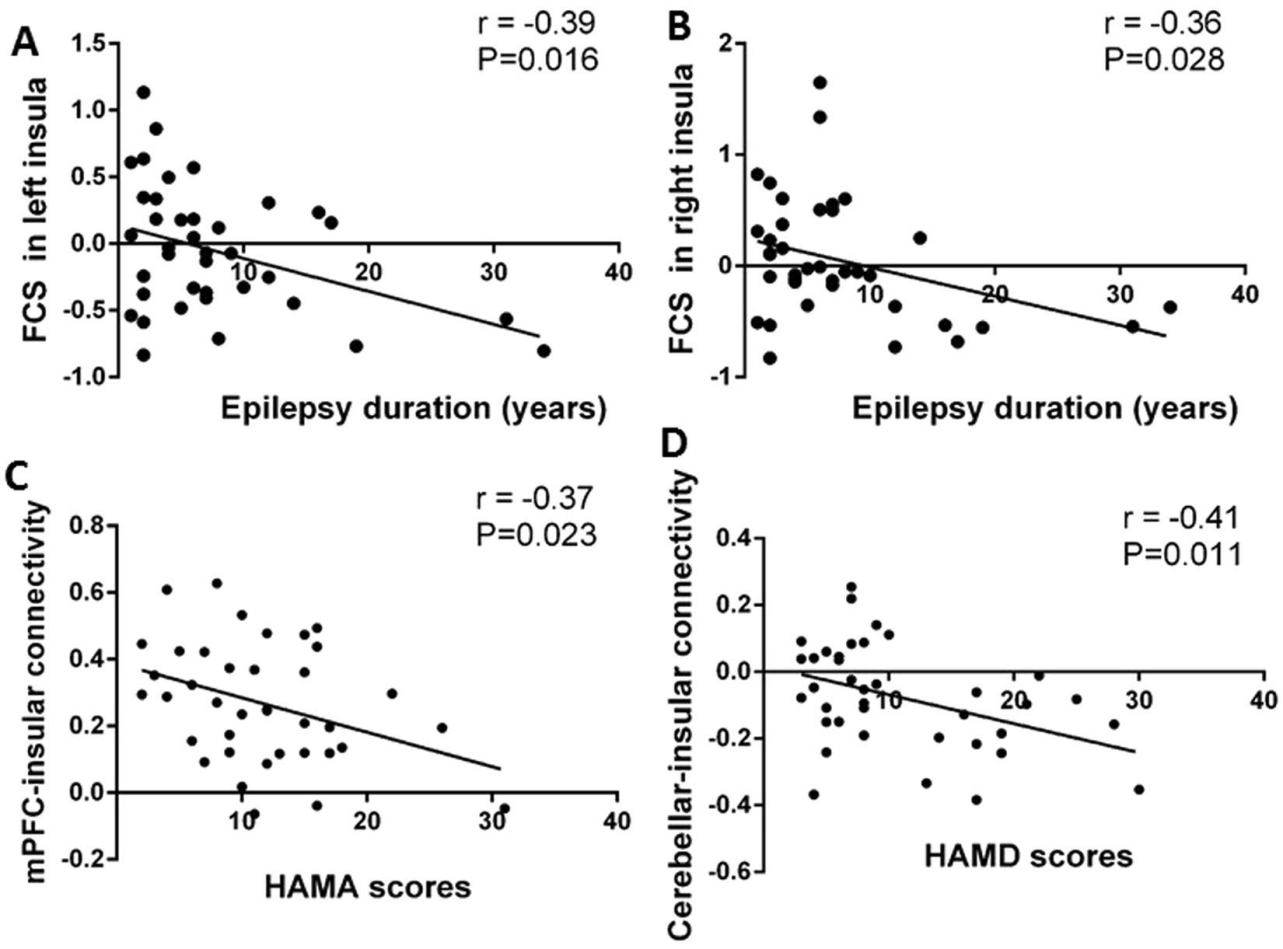
Linear Pearson associations between clinical features of PNH patients and altered functional neural correlates including FCS and FC. Scatter plots show the significant negative correlation between the epilepsy duration and the FCS in the left (**A**) and right insula (**B**). The FC between the left insula and ACC/mPFC was negative associated the HAMA total score (**C**) while the FC between the left insula and left cerebellum was negative associated the HAMD total score (**D**). *Abbreviations:* ACC, anterior cingulate cortex; FC, functional connectivity; FCS, functional connectivity strength; HAMA, Hamilton Anxiety Scale; HAMD, Hamilton Depression Scale; mPFC, medial prefrontal cortex; PNH, periventricular nodular heterotopia.

The linear Pearson correlation coefficients between clinical characteristics and altered FCS as well as FC of PNH patients were calculated. FCS in the left insula was negatively associated with epilepsy duration (r = −0.39, p = 0.016) (Fig. 3A). FCS in the right insula was similarly negatively correlated with epilepsy duration (r = −0.36, p = 0.028) (Fig. 3B). No significant correlations were observed between the frequency of seizures and FCS in bilateral insula.

For PNH-related epilepsy patients, the higher FC between the left insula and the ACC/mPFC was negatively correlated with HAMA scores (r = −0.37, P = 0.023) (Fig. 3C). The higher FC between the left insula and the left cerebellum was also found to be negatively correlated with HAMD scores (r = − 0.41, P = 0.011) (Fig. 3D). There were no significant correlations between the altered FCS values and the HAMA and HAMD test results in participants with PNH-related epilepsy (P > 0.05).

## Discussion

To the best of our knowledge, this is the first study to integrate both whole-brain FC and seed-based FC analyses to explore the network-level neural function alterations in patients with PNH. Additionally, we performed bivariative voxel-wise Granger causality analysis to characterize the dynamic influences between brain regions with significant neural functional alterations. Our study produced three main findings. (1) In comparison with healthy controls, patients with PNH demonstrated lower FCS in bilateral insula, higher seed-based FC within the precuneus and lower connectivity in the ACC/mPFC and cerebellum networks. (2) The GCA results revealed a significant hyper-influence of the left insula over the precuneus of subjects with PNH (3) The decreased functional connectivity was correlated with emotional state assessed by the HAMA and HAMD, pointing to a connectivity-based pathophysiologic process in PNH.

In a previous resting-state fMRI study on PNH, with the seed defined by the area of the precuneus where lower ALFF was detected, patients with PNH showed significantly higher FC in the right insula^11^. This finding was in agreement with the current study. However, another prior resting-state FC study on PNH revealed no FC alterations in insula^7^. The discrepancy may be attributed to the sample size differences between our study and the previous one. We included a larger number of PNH patients in the current study (N = 38) than previous study (N = 11), which increases the statistical power as well as the chance of true possible findings. Moreover, the previous study included nodules as their ROIs, while in our study the FCS without ROI was applied.

PNH induced a disrupted functional connection between the insula and the brain default mode network (DMN), which was widely described as abnormal in epilepsy. Additionally, the same component had a number of structures observed in the DMN in humans, including brain regions of the mPFC, posterior cingulate cortex/precuneus, bilateral temporal–parietal areas, bilateral dorsolateral frontal cortices and cerebellum^18^. It is reported to have greater activity when individuals are in resting-state networks rather than during a task. In the present study, changes in remote functional connectivity values in this sample population occurred in precuneus, mPFC and cerebellum also involving the DMN.

In the current study, we found FCS reductions in the bilateral insular areas and higher FC in the precuneus in patients with PNH relative to controls. The insula is the core structure of the salience network (SN) which plays a key role in regulating the dynamic changes of external stimuli and internal events^19^ while the precuneus is the main component of the default mode network (DMN), which is involved in the monitoring of internal processes including autobiographical and self-monitoring^20^. The hypoactivation of the insula and the hyperactivation of precuneus suggested the disequilibrium between the DMN and the SN might be associated with the pathophysiology of PNH. Furthermore, the GCA results revealed a significant hyper-influence of the left insula over the precuneus of subjects with PNH, indicating that the insula might be the cause of the disease.

The involvement of DMN could be an explanation to the altered state of consciousness during seizure. The precuneus has long been thought to be responsible for monitoring both internal and external environments. Multiple lines of evidence suggest that the precuneus is involved in self-related mental representations during rest and seizure^21,22^. The most common clinical feature of patients with PNH is epileptic seizures, mainly focal seizures without awareness. This provides evidence that the precuneus area is a key ingredient containing functions of consciousness in PNH-related epilepsy.

In the processing of human emotion, the mPFC and ACC were reported to play a pivotal role in observing internal psychological states and regulating both social behavior and emotional expression. The mPFC was also reported to regulate and closely connect with the limbic system, including the ACC and amygdala^23,24^. The present study observed significantly decreased functional connectivity in the ACC/mPFC, indicating that affective network in PNH-related epilepsy was impaired, which may account for the behavioral and emotional comorbidities. This was further confirmed by the correlation between HAMA scores and functional connectivity values of mPFC. The mPFC can be subdivided into different subregions showing various connectivity patterns with regions to adjust the productions of anxiety behaviors and differentially contribute to anxiety. There are also other studies demonstrating the association of mPFC with depression in epilepsy^25–27^, which is in accordance with epileptic mouse model which demonstrated the correlations between behavioral pathogenesis and altered activity of mPFC^28^.

As for depression, it has been reported to be associated with disrupted functional connectivity in frontostriatal-limbic brain networks^29^, and alterations of functional connectivity were also revealed in regions including insula, lateral frontal poles, and the elements of DMN like ventromedial prefrontal cortex prefrontal cortex (vmPFC)^30,31^. Moreover, the cerebellum may also be involved in cognition and affection, as we found the cerebellum was negatively affected in PNH patients, beyond the well-known role in motor domain^32^. Multiple lines of evidence have suggested that the cerebellum and its subregions are related to different neural networks in cognitive and affective processing. In psychiatric or emotional disorders, including depression, autism spectrum disorder, and even schizophrenia, abnormalities including structure and function of the cerebellum have also been explored^33^. Specifically in PNH, we have previously found PNH cases with autism along with cerebellar abnormalities, such as cerebellar dysgenesis and vermisdysgenesis^34^. Additionaly, the histopathological studies showed that the abnormalities and disorganization of cerebellum were closely linked to autism spectrum disorders^35^. Another functional MRI study revealed significantly disrupted regional homogeneity values in regions including the left cerebellum in patients with depression compared to HCs^36^. Another large-scale study assessed brain organization in cortical malformations including heterotopia and found less marked anomalies in heterotopia across subtypes classified by different stages of brain development, suggesting that disturbances of early stage in cortical development may not necessarily result in detrimental effects. Since we only enrolled the subtype of PNH, further studies across subtypes of cortical development are necessary to explore the different patterns of functional connectivity^4^. Taken together, the abnormal activities of cerebellum in the PNH group may implicate the disruption of emotion state.

There are several limitations to our study. First, the number of participants in the study was relatively small. Future investigations with larger sample size would be needed to verify our findings. Second, we could not rule out the effects of medication on our findings since the majority of the PNH patients were treated by antiepileptic drugs. Third, we could not identify the association between the anomalies of functional neural substrates and cognitive deficits in PNH patients because we did not perform the neuropsychological tests for PNH patients to evaluate cognitive function. Forth, although insular seems to be the hub that is identified with FCS, the role of insular needs to be taken with caution as the core of the disease. Finally, since the age of seizure onset for PNH is often between 10–20 years, future studies following the development of these patients from childhood to adults will provide valuable information about the progression of PNH.

Our findings have provided explanation of PNH-related brain network hub changes, both the functional connectivity strength and remote functional connectivity. Using the resting state FCS analytical approach, we identified significant insular hypoactivation in PNH patients, suggesting that the insula might represent the cortical hub of whole-brain networks under this condition. Additionally, disruption of resting state FC in large-scale neural networks pointed to a connectivity-based neuropathological process in PNH. It is helpful in expanding our understanding of migration anomalies in brain development which usually causes epilepsy and even emotional dysfunction. Furthermore, epilepsy and comorbid emotional abnormalities have been drawing increasing attention of public, and our results provide evidence for that the altered functional neural networks may be a possible mechanism underlying seizures and impairment in behavioral function.

## Materials and Methods

### Participants

This study recruited 38 patients diagnosed with PNH and epilepsy from our epilepsy center in the West China Hospital of the Sichuan University. This study was approved by the local ethical committee of the West China Hospital of Sichuan University, and all subjects have provided informed consent. All methods were performed in accordance with the relevant guidelines and regulations. Our inclusion criteria were as follows: (1) at least one visible gray matter nodule detected lining the lateral ventricular walls, on more than one slice of T1 or T2 MR images in the structural MRI of patients; (2) all patients with PNH had demonstrated the presence of seizures in accordance with International League Against Epilepsy report of classification in 2010^37^. Moreover, we excluded any patients with PNH and epilepsy that met any of the following conditions: (1) focal malformation of brain development on conventional T1 or T2-weighted MR images additional to PNH, (2) prior history of brain surgery, (3) head motion of patients >1 mm in translation or 1° in rotation during fMRI scanning, (4) alcohol/drug abuse or (5) history of neurological or psychiatric disease. Eventually a total of 38 patients with PNH and epilepsy, 37 of whom had been treated with antiepileptic drugs, and 1 patient was drug-naïve, were included in this study. Additionally, 38 healthy controls matched for age and sex were also enrolled through posters. The exclusions for HCs were as follows: (1) neurologic or psychiatric disorders, (2) apparently abnormalities on brain MRI, or (3) they were related to one of the enrolled patients with PNH. Moreover, the detailed demographic, clinical, and genetic information including duration of epilepsy and response to treatment were all collected and patients were monitored every three months at the epilepsy center.

### Questionnaires

Examination of mental state was performed by Mini-mental State Examination (MMSE) first. The DSM-IV and Hamilton Rating Scale were employed to evaluate major anxiety and depressive disorder and assess the severity. To evaluate the severity of anxiety and depression, we used Chinese versions of the 14-item Hamilton Anxiety Scale (HAMA) and 24-item Hamilton Depression Scale (HAMD), which had been determined to have satisfied validity and reliability in Chinese people^38^. They were translated from HAMA^39^ and HAMD^40^.

### Data acquisition

All subjects underwent functional MRI scanning using a 3.0 T MRI scanner (Siemens Trio) with an 8 channel phase array head coil. Each subject used the earplugs for reducing the scanner noise. In addition, we used foam padding for minimizing the head motion of each subject. The resting state fMRI sensitized to alterations in BOLD signal levels of the whole brain was obtained via an echo-planar imaging (EPI) sequence. (TR is 2000 ms, echo time is 30 ms, flip angle is 90°, slice thickness is 5 mm, with no slice gap, field of view = 240 × 240 mm^2^, voxel size is 3.75 × 3.75 × 5 mm^3^, 30 axial slices, 200 volumes in each run). During the data collection, all of the subjects were instructed to close their eyes, remain still, and relax, without falling asleep during scanning.

### Data processing

We used the DPABI software^41^ to perform the preprocessing step of the resting-state fMRI scans. Specifically, in order to allow for signal equilibrium and allow the subjects’ adaptation to the noise during scanning, the first 10 volumes of each subject were omitted. The images were then corrected for slice-timing. we adopted the Friston 24-parameter model as a regressor, which has been shown to be advantageous to the 6-parameter model, to remove the head motion artifacts,^42^. All the functional image data used in the present investigation met the criteria of spatial movement in any direction <1 mm or 1 degree and the mean framewise displacement (FD) value < 0.2. Afterwards, all the images were further spatially normalized to the Montreal Neurological Institute (MNI) template, followed by spatially resampled to a voxel size of 3 × 3 × 3 mm^3^. Additionally, signal from the cerebrospinal fluid, white matter and global mean signal intensity were used as covariates. Subsequently, we removed the linear trend of the fMRI images and conduct the band-pass filtering (0.01–0.08 Hz) in order to minimize the effect of physiological noise with high frequency as well as the extreme low-frequency drift. After that, the REST toolbox^43^ was used for the calculation of FCS.

### FCS calculation

The calculation of FCS was performed as described previously^17^. First, the Pearson’s correlations were computed between the time series of all pairs of voxels to construct a whole-brain connectivity matrix for each subject. This step was limited within a mask of gray matter generated in SPM12. Subsequently, we computed the FCS of a given voxel by counting the sum of its weighted connections with all other voxels. Such FCS index is also termed as the “degree centrality” of weighted networks with regard to graph theory. Afterwards, we used Fisher r-to-z transformation to convert individual correlation matrices into a z-score matrix. We adopted the correlation threshold (r = 0.2) in the current FCS calculation in order to eliminate voxels with weak correlations attributable to signal noise and remove negative correlations generated by global signal regression. Finally, we used SPM12 toolbox to smooth the FCS maps with a 6-mm Gaussian kernel before statistical analysis.

### Seed-based FC analysis

We selected the areas with abnormal FCS values in PNH relative to HCS as ROIs to perform an additional seed-based interregional correlation analytical approach in order to explore more specific abnormalities of FC patterns related to the identified hubs. We used the REST software^43^ to perform the seed-based FC analyses. Specifically, after band pass filtering (0.01–0.08 Hz) and linear trend removed for each seed, we extracted reference time series by averaging the rs-fMRI time series of voxels within each seed. Afterwards, the mean time series in each seed was correlated with other voxels across the whole brain. For the purpose of improving the normality, we applied the Fisher r-to-z transformation to converting the correlation coefficients in each voxel into z-scores. Finally, bivariative voxel-wise Granger causality analysis (GCA) implemented REST toolbox was performed to characterize the dynamic influences between brain regions with significant functional alteration^44^.

### Statistical analyses

We adopted two-sample t tests for examining differences regarding the demographic characteristics and clinical features between participants with PNH and control subjects using the SPSS software.

Voxel-wise statistical comparisons of both whole-brain FC alterations (FCS maps) and seed-based FC abnormalities (Z maps) between PNH subjects and controls were conducted with a two-sample t-test in SPM12. The voxel level statistical threshold was set at P < 0.001, with a minimum cluster extent of 100 voxels without correction. Meanwhile, the statistical threshold of cluster level was set at P < 0.05 with family-wise error (FWE) correction. Regions with significant FCS and FC alterations between groups were extracted as ROI for Pearson correlation analyses with clinical features such as the seizure frequency and the duration of epilepsy in the SPSS software.

## Supplementary information

Figure S1

## Data Availability

The datasets generated during and/or analysed during the current study are available from the corresponding author on reasonable request.
