# Supplementary material for: Resting-state functional connectivity alterations in periventricular nodular heterotopia related epilepsy
Source: Sci Rep. 2019 Dec 5;9:18473. doi: 10.1038/s41598-019-55002-3 (PMC6895037; doi:10.1038/s41598-019-55002-3)

**Resting-state functional connectivity alterations in periventricular nodular heterotopia related epilepsy**

Wenyu Liu^1#^, Xinyu Hu^1#^, Dongmei An^1^, Dong Zhou^1*^, Qiyong Gong^2*^

1. Departments of Neurology, West China Hospital, Sichuan University, No. 37 GuoXue Alley, Chengdu, 610041, China;

2. Departments of Radiology, Huaxi MR Research Center (HMRRC), West China Hospital, Sichuan University, No. 37 GuoXue Alley, Chengdu, 610041, China;

# These two authors contribute equally to this article.

* Correspondence:

Dong Zhou, Email: zhoudong66@yahoo.de;

Qiyong Gong, Email: qiyonggong@hmrrc.org.cn.

Figure S1. Granger Causal influence from left insula to precuneus


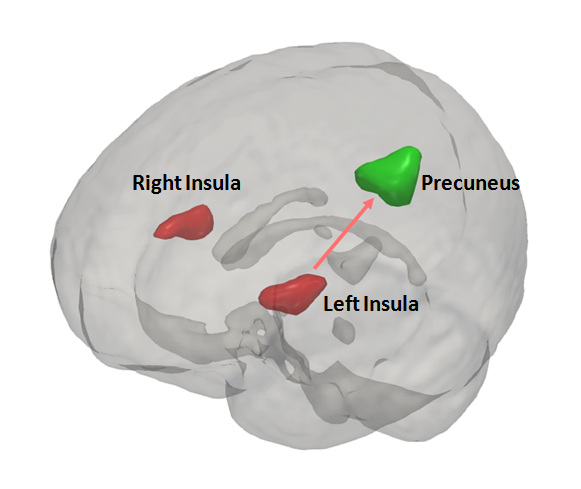

Supplement: Supplementary file 1 — Figure S1 [file 41598_2019_55002_MOESM1_ESM.docx]
